# Aberrant Signaling Pathways in Glioma

**DOI:** 10.3390/cancers3033242

**Published:** 2011-08-10

**Authors:** Mitsutoshi Nakada, Daisuke Kita, Takuya Watanabe, Yutaka Hayashi, Lei Teng, Ilya V. Pyko, Jun-Ichiro Hamada

**Affiliations:** 1 Department of Neurosurgery, Graduate School of Medical Science, Kanazawa University, Kanazawa, Ishikawa 920-8641, Japan; E-Mails: kitad@ns.m.kanazawa-u.ac.jp (D.K.); twatanabe@ns.m.kanazawa-u.ac.jp (T.W.); yuh@ns.m.kanazawa-u.ac.jp (Y.H.); ilia11892@gmail.com (I.V.P.); jhamada@ns.m.kanazawa-u.ac.jp (J.-I.H.); 2 Department of Neurosurgery, The First Clinical College of Harbin Medical University, Nangang, Harbin 150001, China; E-Mail: tengleina@hotmail.com (L.T.)

**Keywords:** glioblastoma, signaling, proliferation, invasion, angiogenesis, molecular target

## Abstract

Glioblastoma multiforme (GBM), a WHO grade IV malignant glioma, is the most common and lethal primary brain tumor in adults; few treatments are available. Median survival rates range from 12–15 months. The biological characteristics of this tumor are exemplified by prominent proliferation, active invasiveness, and rich angiogenesis. This is mainly due to highly deregulated signaling pathways in the tumor. Studies of these signaling pathways have greatly increased our understanding of the biology and clinical behavior of GBM. An integrated view of signal transduction will provide a more useful approach in designing novel therapies for this devastating disease. In this review, we summarize the current understanding of GBM signaling pathways with a focus on potential molecular targets for anti-signaling molecular therapies.

## 1. Introduction

Despite advances in our understanding of the molecular biology of glioblastoma multiforme (GBM), one of the most aggressive human cancers, these tumors are still incurable. GBMs almost universally recur after conventional therapies, including maximal surgical resection, radiation, and chemotherapy, because GBMs are a heterogeneous group of tumors with variable natural history and treatment responses. Recent advances in research techniques have given rise to a wealth of new information regarding the distinct molecular subgroups of GBM with aberrant signaling pathways that drive the tumor phenotype. According to the genomic analysis by The Cancer Genome Atlas (TCGA) network, GBM has four distinct molecular subtypes: Proneural, neural, classical, and mesenchymal [1,2].

The pathogenesis of GBM is complex due to a highly deregulated tumor genome with opportunistic deletion of tumor suppressor genes, amplification, and/or mutational hyper-activation of oncogenes, which are involved in a network of interconnected signaling pathways [2]. The signaling pathway has been described in systems biology terms as a complex biological network of three steps: (1) an input step in which membrane receptors and their ligands trigger the signal coming from outside the cell; (2) a core system processing step in which protein kinases transmit the signal to the nucleus; (3) and an output step in which transcription factors regulate genes that affect various cellular functions. GBM characteristics are derived from the activation of these pathways, including uncontrolled proliferation, invasion, and angiogenesis. Current efforts therefore focus on the development of new molecular approaches targeting these genetic lesions [3,4]. In this review, we provide an overview of the most recent developments and current understanding of GBM signaling and the therapeutic potential of molecular targeted therapy in GBM.

## 2. The Character of Glioma

### 2.1. General Knowledge of GBM

Diffuse astrocytic tumors are the most frequent intracranial neoplasms and account for more than 60% of all primary brain tumors. The major characteristic by which astrocytic tumors are grouped is the histological resemblance of the tumor cells to astrocytes. Therefore, the presence of cells with fine fibrillary processes, expressing glial fibrillary acidic protein (GFAP), is a major diagnostic feature in these tumors. A proportion of the cells demonstrating astrocytic features and GFAP staining is important for identifying the astrocytic tumor cells, even in the more undifferentiated and anaplastic type of tumor. Usually, the tumor cells diffusely infiltrate the adjacent brain parenchyma and are thus termed as “diffuse” astrocytic tumors [5].

According to WHO classification and grading, diffuse astrocytic tumors are subdivided into three categories: (1) diffuse astrocytoma (grade II); (2) anaplastic astrocytoma (grade III); and (3) the most undifferentiated GBM (grade IV). Previously termed “glioblastoma multiforme” because of the heterogeneity of its macroscopic appearance, the name ‘glioblastoma’ is now considered less complicated. GBM is the most malignant astrocytic tumor, composed of poorly differentiated neoplastic astrocytes. The presence of microvascular proliferation and/or necrosis is essential for histo-pathological diagnosis of GBM. Two subgroups of GBMs have been established based on clinical experience and have been affiliated with distinct genetic mechanisms of tumorigenesis, although these two groups are histologically indistinguishable. Primary GBMs seem to develop rapidly and manifest high-grade lesion from the outset. In contrast, secondary GBMs develop slowly through progression from grade II or grade III.

### 2.2. Clinical Features of GBM

#### 2.2.1. Incidence and site

GBMs are the most common primary brain tumors in adult neurosurgical practice, and account for 40%–60% of all diffuse astrocytic tumors and 10%–15% of all intracranial neoplastic lesions. Although they may occur at any age, GBMs have a peak incidence between 50 and 70 years. The cerebral hemispheres are the most common site for GBM, with 95% arising in the supratentorial region, and less commonly appearing in the brain stem, cerebellum, and spinal cord.

#### 2.2.2. Radiology

**Figure 1.**
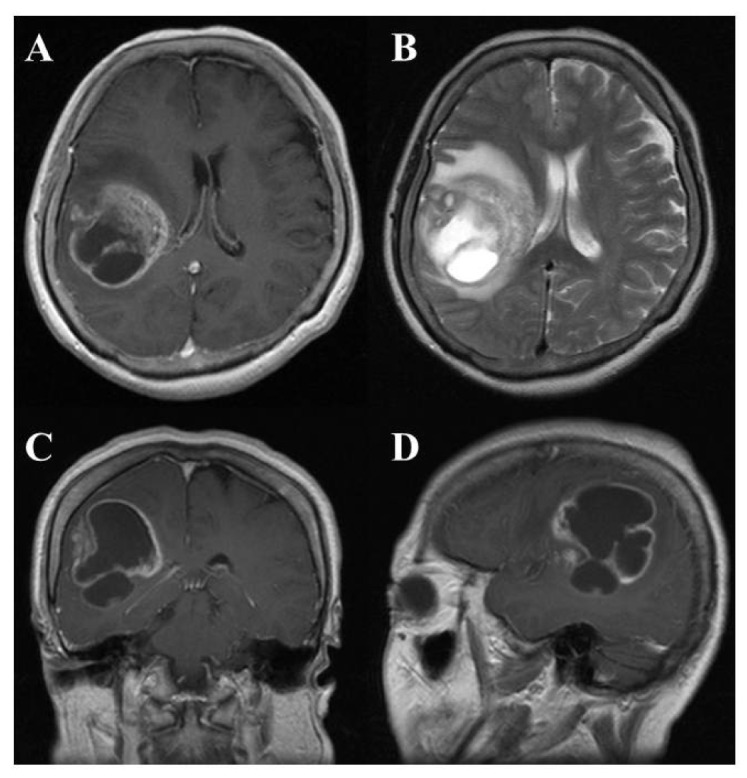
Representative radiological findings of GBM. Gadolinium-enhanced T1-weighted MRI (A, axial section; C, coronal section; D, sagittal section) shows a ring enhancement lesion in the right temporo-parietal lobe with midline shift. T2-weighted (B) image showed prominent peritumoral edema.

On computed tomography (CT) and magnetic resonance imaging (MRI), many GBMs present a typical appearance of ring enhancement indicating a central necrosis surrounded by enhancing viable tumor (Figure 1). Peritumoral edema, usually with distortion of the surrounding brain and ventricles (mass effect), is well visualized by MRI. Despite the typical appearance, other enhancing lesions within the cerebral hemisphere, especially abscess, metastasis, lymphoma, or even acute multiple sclerosis plaques and subacute infarctions, should be considered as notable differential diagnoses.

#### 2.2.3. Therapy and prognosis of GBM

The prognosis for GBM is worse than that for diffuse astrocytoma and anaplastic astrocytoma. Most clinical studies suggest that 50% of patients with GBM will survive no longer than one year. The outcome of GBM depends largely upon the patient's preoperatively scored neurological state, scored by the Karnofsky Performance Status (KPS), age, and tumor site [6,7]. Generally, individuals under the age of 50 have a slightly longer survival time than those over 50, and patients over the age of 65 have a particularly poor prognosis. The location of the tumor is closely related with radical surgical removal and it is an important factor in tumor management. GBMs deeply seated in the thalamus and basal ganglia, and thus eloquent cortices (motor cortex, sensory cortex, language cortex, *etc.*) inaccessible to surgical excision, have a poor prognosis. GBMs in the non-eloquent frontal and temporal lobes, which are accessible to excision, may have a slightly longer survival time. The recent advent of intraoperative surgical modalities including neuronavigation, intraoperative monitoring, and 5-aminolevulinic acid (5-ALA) fluorescence-guided tumor resection, have improved the rate of gross total excision and prolonged survival time [2].

Corticosteroid treatment is often very effective initially in patients with GBM. They reduce intracranial pressure and reduce the severity of symptoms due to early-stage tumors. After surgical resection, radiotherapy also has some beneficial effect on the overall prognosis and may increase the quality of life for the patient; many hospitals employ chemotherapy after/with radiotherapy. In particular, chemotherapy with the recently introduced chemo-agent temozolomide plus radiotherapy has been proven effective on newly diagnosed GBM patients and has provided a new standard of care after tumor excision [8]. However, despite such combined therapy with surgical resection with adjuvant chemo/radiotherapy, overall survival time in GBM patients is still never more than two years.

### 2.3. GBM Cell Biology

**Figure 2.**
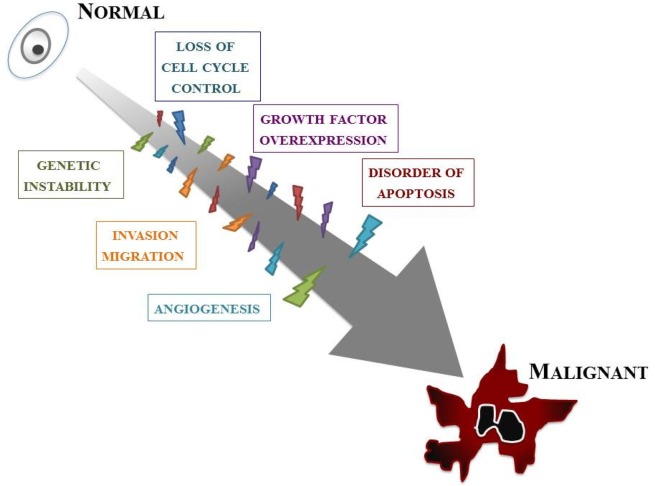
Major events during gliomagenesis. Neoplastic transformation in GBM is attributed to the progressive accumulation of multiple intracellular events.

Our knowledge of the molecular biology of GBM has increased markedly over the last two decades. GBM cells are subjected to many kinds of cellular dysfunction, which make themselves resistant to various anti-GBM therapies. Six typical intracellular events that characterize glioma are overviewed below, but it should be stressed that such events do not arise in isolation; a combination of multiple tumorigenic events cause and sustain GBM (Figure 2).

The first event is loss of cell cycle control. The progression of the normal cell cycle is under strict control. However, glioma cells develop means for evading such control, giving them a growth benefit. The cell cycle checkpoint that has received the most attention is the G1–S phase transition. p16^INK4a^/cyclin-dependent kinase (CDK)-4/RB (retinoblastoma) 1 pathway, one of the major pathways controlling this checkpoint, involves p16, CDK-4, cyclin D1, and RB1 [9]. The CDK/cyclin D1 complex phosphorylates RB1, thereby inducing release of the E2F transcription factor that activates genes involved in the G1/S transition [10]. Many of the genetic defects in growth regulatory molecules occur preferentially in malignant, rather than low-grade, gliomas. Alterations of at least one component of this pathway occur in many anaplastic astrocytomas and in the vast majority of GBMs [11,12]. Nearly all high-grade tumors have impairments of this single critical cell cycle control pathway. It is likely as well that less profound defects in cell cycle regulation occur in low-grade gliomas; for instance, *p53* gene mutations may affect both the G1–S and G2–M checkpoints.

The second event is overexpression of growth factors and their receptors. A variety of growth factors such as epidermal growth factor receptor (EGFR), platelet-derived growth factor (PDGF), basic fibroblast growth factor (bFGF, FGF-2), transforming growth factor (TGF)-α, and insulin-like growth factor (IGF)-1 are overexpressed in GBM and thus provide a growth advantage to neoplastic cells. Generally, glioma cells express both the growth factor ligands and their receptors, setting up an autocrine growth-promoting loop. Of these, EGFR and PDGF have been best characterized in GBMs [13,14]. EGFR is a transmembrane receptor responsible for sensing extracellular ligands, such as EGF and TGF-α, and for transducing this proliferation signal.

Angiogenesis is also an important feature. A dramatic sequence of angiogenic alterations occurs in the progression to GBMs, which in MRI appears as ring-like contrast enhancements that surround rapidly growing tumors [15]. Malignant gliomas are vascular tumors, and the histological presence of microvascular proliferation indicates a high grade. Angiogenic molecules have been found in malignant gliomas, primarily in GBMs [16]. Vascular endothelial growth factor (VEGF) is the most clearly implicated and an endothelial cell mitogenic factor that is expressed most often adjacent to areas of necrosis but not in low-grade astrocytomas. This suggests that the malignant progression from low-grade astrocytomas to GBMs includes an “angiogenic switch”.

One of the key features of GBMs, invasion and migration, is their diffuse infiltration of the surrounding neural net. The expression of several extracellular matrix (ECM) molecules and cell surface receptors may modulate signal transduction and influence invasion and migration in GBMs [5,17,18]. These include cytoskeletal proteins; signaling molecules that mediate interactions between the microenvironment and the cytoskeleton; cell surface receptors involved in cell migration such as transmembrane adhesion molecules; and components of ECM, including proteases.

Abnormality of apoptosis should be mentioned. Apoptosis refers to cell death that occurs in a programmed manner, characterized by non-inflammatory cellular condensation. Glioma cells may develop means not only for increasing proliferation but for abrogating apoptosis as well. A number of genes involved in gliomagenesis have roles in apoptosis, most notably *p53* [19,20]. *p53* mutations disturb the normal glial apoptotic response that would follow growth factor overexpression in low-grade gliomas, allowing further progression [21].

The last intracellular event is genetic instability. An essential feature of low-grade gliomas is their nearly universal progression to higher-grade lesions over time. Such malignant progression is related to the emergence of more malignant clones. Genomic instability, a feature of many tumors, encourages further genomic damage, thus allowing the eventual selection of more malignant clones [22]. Mutations in *p53*, also known as “guardian of the genome”, may therefore lead to tumor progression through genomic instability. Patients with syndromes of genomic instability, such as the Turcot syndrome, have an increased susceptibility to malignant gliomas [23].

## 3. Proliferation Signaling

**Figure 3.**
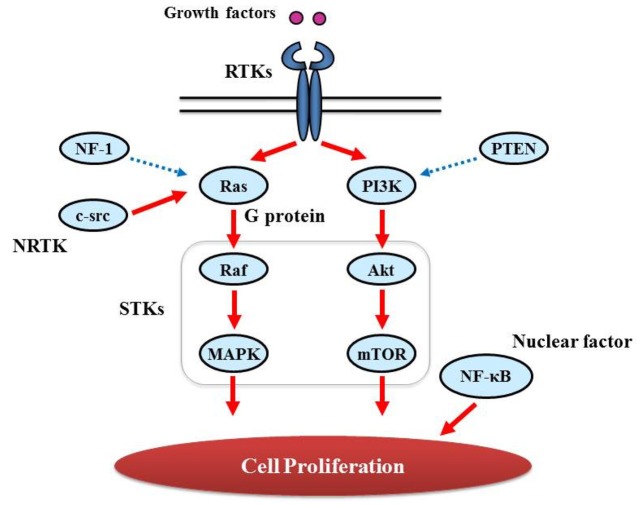
Main proliferation signaling pathways. The PI3K/Akt/mTOR and Ras/Raf/MAPK pathways are shown. Growth factors bind to Receptor tyrosine kinases (RTKs), followed by activation of Ras/Raf/MAPK and/or PI3K/Akt/mTOR. Raf, MAPK, Akt, and mTOR are classified as serine/threonine-specific protein kinases (STKs). Intracellular tyrosine kinase c-src also activates Ras/Raf/MAPK. Nuclear factor NF-κB also plays an important role in cell proliferation. Ras/Raf/MAPK and PI3K/Akt/mTOR signaling pathways are depicted in detail in Figures 6 and 8, respectively. Solid and dashed arrows indicate activation and suppression, respectively. c-src: v-src sarcoma (Schmidt-Ruppin A-2) viral oncogene homolog (avian); MAPK: Mitogen-activated protein (MAP) kinase; mTOR: mammalian target of rapamycin; NF-κB: nuclear factor kappa-light-chain-enhancer of activated B cells; NF-1: neurofibromin 1; NRTK: non-receptor tyrosine kinase; PI3K: phosphatidylinositol 3-kinases; PTEN: phosphatase and tensin homolog.

Proliferative activity with histopathologically detectable mitoses is prominent in almost all GBM cases. It is essential to define the contribution of proliferation signaling (or growth factor-mediated signaling) to the GBM cellular phenotype in order to understand the biology of transformation in the CNS. Two of the most important signaling cascades frequently deregulated in glioma are the PI3K/Akt/mTOR and Ras/MEK/MAPK pathways (Figure 3).

### 3.1. Tyrosine Kinase (TK) and Its Ligand

In glioma growth and progression, proliferation signaling depends largely upon the activity of cell surface membrane receptors that control the intracellular signal transduction pathways regulating proliferation; in other words, the leading actor is tyrosine kinase.

#### 3.1.1. Receptor Tyrosine Kinase (RTK)

RTKs are glycoproteins that possess an N-terminal extracellular ligand-binding domain, a single anchoring transmembrane α helix, and a cytosolic C-terminal domain that contains the catalytic domain [24]. For instance, EGFR is a transmembrane glycoprotein member of the ErbB receptor family. In response to binding of ligands EGF and TGF-α, the receptor homodimerizes and/or forms heterodimers with other similar but not identical kinases of the same subfamily, especially ErbB-2 (HER2), allowing a highly varied response to the extracellular signal [25,26]. RTK activation in glioma can be achieved through protein overexpression or genetic amplification of several RTKs, and through gene mutations that lead to constitutive activity. In GBM, EGFR is dysregulated through overexpression, which arises because of EGFR gene amplification or activating mutations such as EGFRvIII that lead to ligand-independent signaling; this is the case in half of all GBM patients [27,28]. Increased transcription of PDGFRα has been observed in astrocytic tumors, but gene amplification is detected only in a limited subset of GBMs (13%) [29,30]. This also creates autocrine or paracrine loops that promote tumor cell proliferation. These receptors are described in detail in “Major signaling and related molecules”. Other RTKs such as FGFR, IGFR, and mesenchymal-epithelial transition factor (c-Met) are abnormally expressed in GBMs [31-34]. Although the mechanisms for such growth factor abnormality, mainly overexpression, vary between growth factors, they are thought to affect glioma proliferation or transformation.

#### 3.1.2. Non-RTK (NRTK)

NRTKs lack transmembrane domains and are found at various intracellular locations, including the cytosol, nucleus, and the inner surface of the plasma membrane. c-src [v-src sarcoma (Schmidt-Ruppin A-2) viral oncogene homolog (avian)] TK includes SH2, SH3, and TK domains. c-src mediates intracellular signaling pathways that control key biologic/oncogenic processes. Increased c-src activity in GBM has been reported [35].

#### 3.1.3. Growth factor

It is well known that various kinds of growth factors, such as PDGF (PDGFA, PDGFB), bFGF (bFGF, FGF-2), TGF (TGF-α, TGF-β), and IGF-1 are overexpressed in GBM [36-39]. However, their role in GBM progression is not always fixed. For instance, TGF-β acts as a tumor suppressor in normal epithelial cells and early-stage tumors and becomes an oncogenic factor in advanced tumors [40].

### 3.2. Others

Molecules that play important roles in GBM proliferation, other than RTK and its ligands, are described below.

#### 3.2.1. GTPase activating protein (G protein)

*Ras* genes, an abbreviation of Rat Sarcoma, encode a protein family of small GTPases that cycle between inactive GDP-bound and active GTP-bound conformations by coupling cell membrane growth factor receptors such as EGFR; they regulate cellular signal transduction by acting as a one-way switch for the transmission of extracellular growth signals to the nucleus [41]. Because these signals result in cell growth and division, dysregulated Ras signaling can lead to oncogenesis and cancer [42]. Ras activates a number of signaling pathways that include stem cell factor (SCF)/c-kit signaling, mammalian target of rapamycin (mTOR), and mitogen-activated protein (MAP) kinases pathways. The role of Ras in GBM is described in detail in “Major signaling and related molecules”.

#### 3.2.2. Serine/threonine specific protein kinase (STK)

The protein kinase family of enzymes plays a pivotal role in signal transduction across the cell membrane as well as inside cells [43]. Both types of protein kinases, TK and STK, share a high sequence similarity in their catalytic domains, which suggests that they descended from a common ancestral gene. STK have received comparatively little attention, vis-a-vis TKs, in terms of their involvement in cancers [44]. In contrast to the TKs, most of the known STKs are cytoplasmic proteins.

STK belongs to the family of transferases, specifically those that transfer phosphorus-containing groups as protein-serine/threonine kinases. STK expression is altered in various cancers. Akt is one of the STKs that play a key role in cellular proliferation. GBM frequently contains alterations in PTEN (Phosphatase and Tensin Homolog Deleted from Chromosome 10) [45] that is a lipid phosphatase and lead to activation of the Akt/mTOR pathway [46]. BRAF, v-RAF murine sarcoma viral oncogene homolog B1, is also an STK and a member of the RAS/RAF/MEK (MAPK kinase)/MAPK pathway that is commonly activated by somatic point mutation V600E in pilocytic astrocytoma [47]. In contrast, this mutation is rarely reported in GBM [48].

TGF-β receptor is predicted to receptor-type STK [49], albeit most growth factor receptors are transmembrane TKs or are associated with cytoplasmic TKs, and plays an important role in growth and progression of GBM, via Smad phosphorylation [50].

#### 3.2.3. Nuclear Factor

NF-κB, nuclear factor kappa-light-chain-enhancer of activated B cells, is a protein complex that controls DNA transcription and regulates genes that control cell proliferation and cell survival [51]. Aberrant constitutive activation of NF-κB in GBM in response to PDGF overexpression promotes glioma cell proliferation [51]. The PI3K pathway has been implicated in mediating the activation of NF-κB by PDGF, and inactivation of PTEN and PDGF expression may contribute to the high levels of NF-κB [52].

## 4. Angiogenesis Signaling

**Figure 4.**
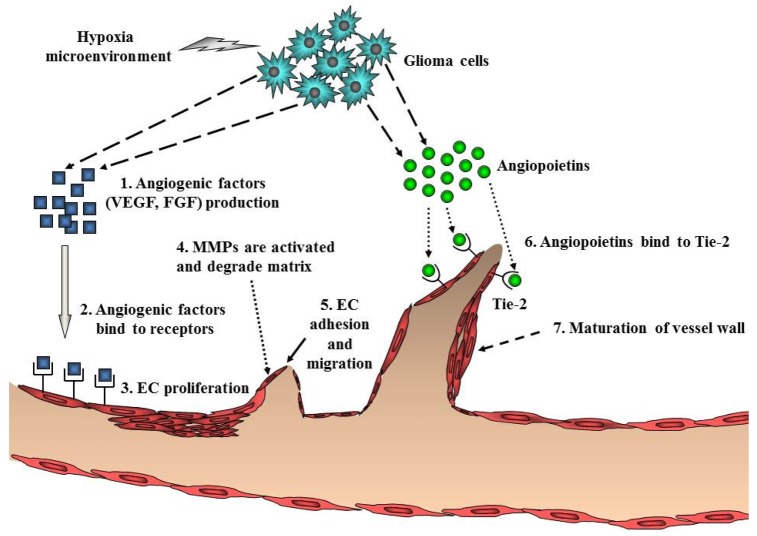
A schematic diagram of angiogenesis in gliomas. Angiogenesis is initiated by angiogenic factors, which are released from glioma cells in the hypoxic glioma microenvironment. Major angiogenic factors include VEGF and FGF. Upon binding to their cognate receptors on endothelial cells, angiogenic factors trigger endothelial cell proliferation and migration. After degradation of ECM, endothelial cells are assembled into a tubular lumen. The final process is maturation of the vessel wall, which is constructed by recruitment of pericytes to assemble along the endothelial cells outside the new vessel.

Ubiquitous angiogenesis is a hallmark of GBMs [53]. The degree of vascularization is significantly correlated with glioma malignancy, tumor aggressiveness, and clinical prognosis [54]. A number of scientists have aggressively investigated angiogenesis signaling and the function of angiogenic factors in malignant glioma. Many studies have implicated pro-angiogenic pathways including a sequence of coordinated events that is initiated by the expression of angiogenic factors such as VEGF with subsequent binding to its cognate receptors on endothelial cells (Figure 4). VEGF/VEGFR (VEGF receptor) participates in the formation of primitive blood vessels from progenitors, hemangioblasts/angioblasts, and the further development of blood vessels in glioma tissue [55,56]. Many other factors increase VEGF expression, including acidosis, nitric oxide, altered oncogenes, and tumor suppressor genes, cytokines such as bFGF, PDGF, and EGF, and activated intracellular signaling pathways such as PI3K/Akt and Ras/MAPK [57] (Table 1). Indeed, increased levels of VEGF and VEGFR in GBM have been reported [58,59].

Endothelial cell migration and proliferation are triggered by VEGF binding to its cognate receptors on endothelial cells [60]. Endothelial cells near the tumor strongly express VEGFR2, which establishes a paracrine signaling loop that stimulates the growth and proliferation of endothelial cells. Local degradation and breakdown of ECM occurs simultaneously, paving the way for newly sprouting vessels. Then, angiopoietins (Ang) are involved in the stability and maintenance of the tumor vasculature. Binding of Ang-2 to its cognate receptor, Tie-2, serves to destabilize vessels, which is a requirement for angiogenesis of glioma [2] (Figure 4). Another key angiogenic process involves cathepsin B and matrix metalloproteinases (MMPs), as well as the expression of matrix proteins such as fibronectin, laminin, tenascin-C, and vitronectin [61]. Formation of the new blood vessel is accomplished by endothelial cells that form a lumen. Individual sprouts are then connected to form vascular loops, through which blood begins to flow. Maturation of the vessel wall then begins by recruitment of pericytes to assemble along the endothelial cells outside the new vessel. The angiogenic process of glioma is completed by the formation of a new basement membrane [62].

## 5. Invasion Signaling

GBM is highly invasive by nature but does not metastasize to other organs like carcinomas in other organs, whereas metastatic brain tumors from other organs usually do not exhibit invasive behavior in the brain. Migrating glioma cells tend to move along the vessels, dendrites, and fibers in white matter. These characteristics suggest that GBM possesses specific biological mechanisms that mediate its invasive nature. The highly infiltrative nature of human gliomas recapitulates the migratory behavior of glial progenitors during development of the CNS, suggesting that the activators, receptors, and signaling proteins that contribute to neural crest cell migration are key players in glioma invasion. Glioma invasion is a complex process involving (1) detachment from the original site; (2) adhesion to the ECM; (3) remodeling of the ECM; and (4) cell migration [5]. Many molecules are involved in each step.

### 5.1 Membrane Type Protein

**Figure 5.**
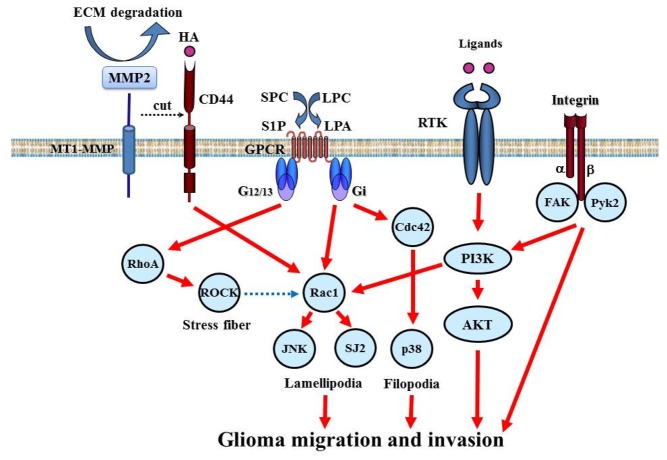
Novel signaling influences glioma invasion. The main signaling pathways involve PI3K/Akt and small GTPases (Rac1, cdc42, RhoA). Solid and dashed arrows indicate activation and suppression, respectively. FAK: focal adhesion kinase; Gi: inhibitory G-protein; GPCR: G-protein-coupled receptor; HA: hyaluronic acid; JNK: c-Jun terminal kinase; LPA: lysophosphatidic acid; LPC: lysophosphatidylcholine; MT1-MMP: membrane-type1 MMP; Pyk2: proline-rich tyrosine kinase; ROCK: Rho-kinase; SJ2: synaptojanin2; SPC: sphingosylphosphorylcholine; S1P: sphingosine-1-phosphate.

Accumulating studies have shown that invasion signaling is induced by several kinds of membrane-type protein such as RTK, integrin, CD44, and G protein-coupled receptor (GPCR, Figure 5).

#### 5.1.1. RTK

RTKs such as EGFR, PDGFR, c-Met, Tie, DDR1 (discoidin domain receptor), Eph, and Axl are expressed at very high levels in GBM and play a major role in glioma invasion [5,63]. Multiple RTKs are simultaneously activated in GBM cells and accelerate invasion signaling. RTK signaling links to intracellular invasion signaling. Additionally, EGFR, Tie, and DDR1 signaling induce the expression and activation of MMP-2, which degrades and remodels the ECM surrounding tumor cells [64-66].

#### 5.1.2. Integrin

Integrins are transmembrane heterodimer receptors consisting of α and β subunits. The combination of α and β subunits determines ligand specificity. Integrins serve to create a link between the ECM and the cytoskeleton actin filaments. This is integral to the maintenance of cell shape and architecture and the regulation of cell migration. Among the 8 β-subunit members, β1 is important to glioma biology and its expression has been correlated with the invasive behavior of glioma [67]. Integrin β5 expression is correlated with *in vitro* invasiveness and migration of glioma cells [68]. Although the β1 subunit can collaborate with several α subunits, α3β1 is consistently over-expressed and is a key regulator of glioma cell migration [69].

#### 5.1.3. CD44

CD44 is a transmembrane glycoprotein expressed in glioma and serves as a surface receptor for components of the ECM such as hyaluronic acid (HA). The standard form of CD44 consists of a cytoplasmic tail, a transmembrane region, and a large (90 amino acid-long) extracellular domain. Under physiological conditions, CD44 in tumor cells is cleaved by a membrane-associated MMP at the membrane-proximal region of the ectodomain. This shedding of CD44 plays a critical role in efficient cell detachment from the HA substrate and promotes cell migration. Overexpression of CD44 in glioma is related to invasion [70]. CD44 can be cleaved by ADAM (a disintegrin and metalloproteinase) 10 and 17, and both the extracellular and intracellular cleaved components of CD44 promote cell migration [71,72]. The CD44 cleavage product is detected in 60% of gliomas. Rac1 activation is linked to CD44 shedding [73].

### 5.2. Intracellular Signaling Molecules

Many invasion signaling pathways converge on the common intracellular signaling molecules PI3K/Akt and small GTPases, such as Rac1, cdc42, and RhoA (Figure 5).

#### 5.2.1. Small GTPases

Small GTP-binding proteins of the Rho family are central regulators of dynamic reorganization of the actin-based cytoskeleton and are key mediators of several cellular processes, including cell migration and polarity [74]. Rac belongs to the Rho GTPases, a family of proteins best known for their regulation of the cytoskeleton and cell migration. Rac1 play key roles in regulating actin polymerization and promoting lamellipodial formation at the front edges of migrating cells. Rac1 is ubiquitously expressed in various tissues. In glioma cells, depletion of Rac1 expression by small interfering RNA reduces cell migration and invasion and strongly inhibits lamellipodia formation [75]. In contrast, Rho activation has been associated with stress fiber formation. RhoA plays a key role in the regulation of actomyosin contractility. Activated Rho relays extracellular signals to a number of downstream effectors, which include Rho kinase (ROCK). Inhibition of ROCK induces glioma cell migration through Rac1 activation, suggesting that the relative degree of activation between RhoA and Rac1 regulates glioma cell movement [76]. Cdc42 plays a critical role in filopodia formation and cell polarity in most eukaryotic organisms. The function of cdc42 in glioma invasion is not completely understood.

## 6. Crosstalk

These signaling elements can contribute to proliferation, survival, migration, invasion, and angiogenesis in GBM cells. A hallmark of signaling networks is the presence of crosstalk, and multiple cross talks can cooperate during glioma initiation and progression to aggressive, invasive, and hyper-vascularized disease stages.

TKs, which are major inducers in GBM, exhibit crosstalk. The c-Met receptor is strongly phosphorylated as a function of EGFRvIII receptor levels, suggesting the presence of crosstalk between c-Met and EGFRvIII signaling, although the intermediary molecule has yet to be elucidated [77]. Axl RTK also follows a similar phosphorylation response as a function of EGFRvIII levels [77]. EGFR and EphA2 RTK are expressed in GBM and co-localize to the cell surface. EphA2 phosphorylation is dependent on EGFR activity and EphA2 downregulation inhibits EGFR phosphorylation, downstream signaling, and EGF-induced cell viability [78].

Inactivation of either Ras/Raf/MAPK or PI3K/Akt/mTOR triggered activation of the other, suggesting that there may be mutually inhibitory crosstalk between them [79] (Figure 3). Generally, Ras/Raf/MAPK is the dominant signaling pathway associated with proliferation, whereas PI3K/Akt/mTOR is the invasion control pathway. The experimental evidence indicates that there may be an inherent and inverse correlation between cell motility and proliferation of a cell population. Consistent with this observation, cDNA microarray data showed that stimulated migration is accompanied by a concomitant down-regulation of genes responsible for proliferation *in vitro* [80].

Although the signaling mechanisms underlying these complex crosstalk phenomena remain to be elucidated, recent studies suggest that signaling crosstalk in GBM may aid in the design of treatment protocols targeting dominant signaling pathways that are preferentially activated.

## 7. Major Signaling Pathways and Related Molecules

### 7.1. RTK/PI3K/Akt Pathway

**Figure 6.**
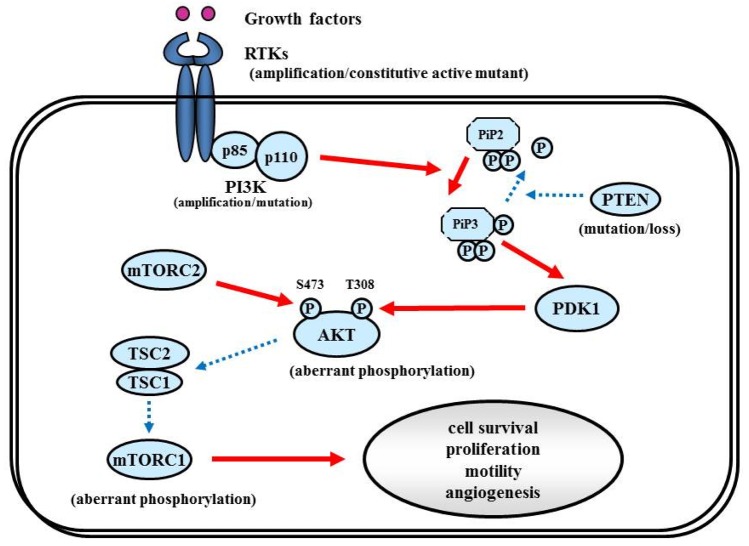
RTK/PI3K/Akt signaling. The binding of RTK, including EGFR and PDGFR, by the p85 subunit of PI3K results in activation of the catalytic subunit (p110), which then catalyzes phosphorylation of PI 3,4-bisphosphate (PiP2) into 3,4,5-triphosphate (PiP3). Inversely, PTEN turns PiP3 into PiP2. PiP3 in turn activates phosphoinositide-dependent kinase-1 (PDK1), which phosphorylates Thr308 of Akt, while Ser473 of Akt is phosphorylated by mTORC2. Activated Akt then inactivates TSC1/TSC2 suppressor complex, which in turn activates mTORC1 as a result. The signaling pathway affects multiple cellular processes including cell survival, proliferation, and motility. Solid and dashed arrows indicate activation and suppression, respectively.

The RTK/PI3K/Akt pathway regulates various cellular processes such as proliferation, growth, apoptosis, and cytoskeletal rearrangement. The pathway involves RTKs (EGFR, PDGFR, and VEGFR, *etc.*) as well as tumor suppressor protein phosphatase PTEN, and protein kinases PI3K, Akt, and mTOR (Figure 6). Aberrant activation of RTK/PI3K/Akt is frequently observed in malignant gliomas because of the alterations of these proteins [81].

#### 7.1.1. RTK

Among all the RTK alterations, *EGFR* gene amplification is the most frequent alteration (approximately 40%) in GBM [82-84]. Amplification of the *EGFR* gene is also associated with structural alteration, and the most common is called EGFRvIII, a mutant that can send ligand-independent constitutive growth signals [13,85,86]. Some reports have shown that GBM patients with EGFR overexpression or mutants have shorter survival, suggesting that alterations of EGFR may be correlated with increased aggressiveness of GBM [87-89]. According to TCGA, *EGFR* aberrations are correlated with a classical subtype of GBM [1,90]. Despite the high frequency of amplification and rearrangement of the *EGFR* gene, EGFR inhibitors (e.g., Gefinitib, Erlotinib) have not elicited clinical responses in patients with GBMs in clinical trials [91-93].

Overexpression of PDGFR (especially PDGFR-α) and PDGF has been observed in astrocytic tumors of all grades, possibly associated with malignant progression [29,36,94,95]. *PDGFRA* amplification (14%), as well as *IDH1* mutation, is a hallmark of the proneural subtype of GBM according to the TCGA consortium, suggesting the association of this subtype and secondary GBM [1,90]. Despite deep association of this molecule with GBM, anti-PDGFR therapy using Imatinib yields only limited clinical responses [96,97].

#### 7.1.2. PI3K

The PI3Ks are widely expressed lipid kinases that promote diverse biological functions. The binding of PI3Ks and RTKs results in activation of Akt through PiP3 and PDK1, which affects multiple cellular processes including cell survival, proliferation, and motility [98] (Figure 6). The PI3K complex is comprised of a catalytically active protein, p110α, encoded by *PIK3CA*, and a regulatory protein, p85α, encoded by *PIK3R1*. Oncogenic mutations or gene amplification of *PIK3CA* has been reported in various neoplasms including human brain tumors [99]. In primary GBM, *PIK3CA* mutations and amplification are observed in 5% and 13% of cases [81]. Unlike *PIK3CA*, *PIK3R1* has rarely been reported as mutated in cancers. The TCGA analysis revealed that *PIK3R1* mutations were found in 10% (9/91) of GBM cases. According to the integrated genomic classification of GBM, *PI3K* mutations (15%) are associated with the proneural subtype [1,90]. First generation of the PI3K inhibitors, LY294002 and wortmannin were not applied for clinical trials because of problems in solubility, selectivity, and toxicity [100]. To date, lots of PI3K inhibitors (many of them are PI3K/mTOR dual inhibitors) such as XL147, XL765 (Exelixis and Sanofi-Aventis), BKM120, BEZ235, BGT226 (Novartis), GDC0980 (Genentech), PKI587, PF04691502 (Pfizer), GSK2126458 (Glaxo-Smith-Kline) are currently undergoing Phase I/II trials for GBM patients.

#### 7.1.3. PTEN

Decreased PTEN activity can activate the RTKs/PI3K/Akt pathway since PTEN negatively regulates the pathway by antagonizing PI3K function [101]. Decreased expression of PTEN and activation of Akt have been shown in a variety of cancers, including GBM [102]. Homozygous deletion or mutation of *PTEN* is a common genetic feature in GBM (∼40%) [1,103,104], resulting in constitutive activation of the RTKs/PI3K/Akt pathway. Some reports implied that GBMs with EGFRvIII and intact PTEN are more likely to respond to EGFR inhibitors [105,106]. PTEN loss is associated with both classical and mesenchymal subtypes of GBM, according to the TCGA study [1].

#### 7.1.4. Akt

Akt is an STK that regulates cell growth, proliferation, and apoptosis. Akt activation has been reported in approximately 80% in human GBMs [46,105], well correlated with the fact that RTKs/PI3K/Akt signaling is altered in 88% of GBM [1]. Oncogenic *Akt* mutations have not been detected in GBM [107]. Akt inhibitor perifosine is undergoing clinical evaluation in malignant gliomas (NCT00590954) [108].

#### 7.1.5. mTOR

mTOR is an STK downstream from Akt. mTOR can be activated by the Akt and RAS pathways. Rapamycin (sirolimus; Wyeth Pharmaceuticals) and its synthetic analogs Torisel (temsirolimus, Wyeth) and Afinitor (everolimus, Novartis), have been intensively evaluated in clinical trials of recurrent malignant gliomas, demonstrating modest efficacy [109,110].

### 7.2. p53

#### 7.2.1. p14^ARF^/MDM2/p53 pathway

**Figure 7.**
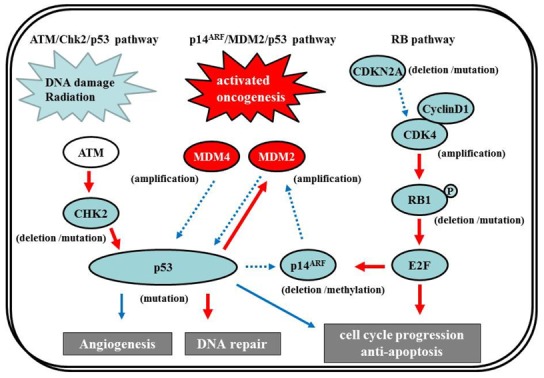
The p53 pathway and retinoblastoma (RB) tumor suppressor protein signaling are shown. MDM2 and MDM4 are important negative regulators of the p53. p14^ARF^ inhibits MDM2, thus promoting p53. DNA damage activates ATM, which leads to activation of checkpoint kinases (CHK2) and p53. Solid and dashed arrows indicate activation and suppression, respectively. Activating genetic alterations are shown in red circle. Genetic alterations that lead to a loss of function are indicated in blue circle. Figure is modified from [121].

The *p53* gene, at chromosome 17q13.1, encodes a protein that responds to diverse cellular stresses to regulate target genes that induce cell cycle arrest, cell death, cell differentiation, senescence, DNA repair, and neovascularization [111]. Following DNA damage, p53 is activated and induces transcription of genes such as p21Waf1/Cip1 that function as regulators of cell cycle progression at G1 phase [10,112,113]. The *MDM2* gene, at chromosome 12q14.3-q15, encodes a putative transcription factor and enhances the tumorigenic potential of cells when it is overexpressed. Forming a tight complex with the *p53* gene, the *MDM2* oncogene inhibits p53 transcriptional activity by binding to the N-terminal transactivation domain, and participates in the ubiquitination and proteasomal degradation of p53 [114,115]. Conversely, the transcription of the *MDM2* gene is induced by wild-type p53 [116,117]. This autoregulatory feedback loop regulates the expression of MDM2 and the activity of p53. The *p14^ARF^* (a part of the complex CDKN2A locus on chromosome 9p21) gene encodes a protein that directly binds to MDM2 and inhibits MDM2-mediated p53 degradation and transactivational silencing [113,118-120]. In turn, p14^ARF^ expression is negatively regulated by p53 [113]. Thus, inactivation of p14^ARF^/MDM2/p53 is caused by altered expression of any of the p53, MDM2, or *p14^ARF^* genes (Figure 7).

The p53 pathway plays a crucial role in the development of secondary GBMs. *IDH1* gene mutations at chromosome 2q33, which were identified in an analysis of 20,661 protein-coding genes in GBMs [1], are early events in the development of astrocytomas and constitute a remarkably reliable molecular signature of secondary GBMs as well as their precursor lesions [122-124]. The additional acquisition of a *p53* mutation, which is a genetic event subsequent to the *IDH1/2* mutations except in the case of Li-Fraumeni syndrome [125], may lead to astrocytic differentiation, while subsequent loss of 1p/19q favors the acquisition of an oligodendroglial phenotype [123,126]. GBMs with *IDH1/2* mutations frequently have *p53* mutations, which are significantly associated [122]. *p53* mutations are also common in two-thirds of precursor low-grade diffuse astrocytomas; this frequency is similar to that in anaplastic astrocytomas and secondary GBMs [28,84,127]. *p53* mutations also occur in primary GBMs, but at a significantly lower frequency (approx. 25%) [128]. In secondary GBMs, 57% of *p53* mutations are located in the hotspot codons 248 and 273; however, in primary GBMs, mutations are more equally distributed through all exons, with only 17% occurring in codons 248 and 273. The less-specific pattern of *p53* mutations may constitute, at least in part, secondary events owing to increasing genomic instability during tumor development.

The *p53* gene is the most commonly mutated *p53* pathway gene in glioma; however, molecular abnormalities involving other genes in the pathway—Such as p14^ARF^, MDM2, or MDM4—have also been described. *MDM2* amplification is observed in about 10% of GBMs, exclusively in primary GBMs that lack a *p53* mutation [12,129]. Loss of *p14^ARF^* expression is frequently present in GBMs. *p14^ARF^* appears to be associated mostly with aberrant promoter methylation or hemizygous deletion, whereas mutational inactivation is rare [130,131]. Promoter methylation of *p14^ARF^* is more frequent in secondary than primary GBMs, but there is no significant difference in the overall frequency of *p14^ARF^* alterations between GBM subtypes [131].

#### 7.2.2 ATM/Chk2/p53 pathway

Recently, in addition to the p14^ARF^/MDM2/p53 pathway, the ATM/Chk2/p53 pathway has come under the spotlight. Squatrito *et al.* reported that loss of the ATM/Chk2/p53 pathway components accelerates glioma development and contributes to radiation resistance [132]. In response to ionizing radiation, cells activate the sensor kinases ATM, ATR, and DNA-PK, a DNA-dependent protein kinase [133,134] that phosphorylates multiple downstream mediators, including the checkpoint kinases Chk1 and Chk2 [135,136], resulting in cell-cycle checkpoint initiation and/or apoptosis. Chk2, at chromosome 22q12.1, can regulate p53-dependent apoptosis in an ATM-independent manner as a tumor suppressor [137,138] (Figure 7). Although previous studies reported no or low frequency of *Chk2* mutations (approx. 6%) [139,140], 22% of glioma patients in the TCGA study presented single-copy loss of the chromosomal region containing *Chk2*, with a significant reduction of Chk2 mRNA, suggesting that it might represent an important tumor suppressor in a subset of glioma patients [1,132].

### 7.3. RB Pathway

The RB pathway suppresses cell cycle entry and progression, as well as the p53 pathway. The 107-kDa RB1 protein encoded by *RB1* (at 13q14) controls progression through G1 into the S-phase of the cell cycle [9]. The CDKN2A (p16^INK4a^) protein binds to CDK4 and inhibits the CDK4/cyclin D1 complex, thus inhibiting cell cycle transition from G1 to S phase (Figure 7) [10,12]. Thus, alteration of *RB1*, *CDK4*, or *CDKN2A* can cause dysregulation of the G1-S phase transition. Inactivation of this pathway is commonly observed in both primary and secondary GBMs [12]. Genetic loss of *RB1* (40%), homozygous deletion of *CDKN2A* (40%), and/or *CDK4* amplification (15%) are detected in the majority of GBM (80%) [11,141], and appear to be roughly mutually exclusive [11,12,142-144]. The TCGA pilot project revealed that the frequency of genetic alterations in the RB signaling pathway was 77%, containing *CDKN2A* homozygous deletion or mutations (52%), *CDKN2B* (p15^INK4b^) homozygous deletion (47%), *CDKN2C* (p18^INK4c^) homozygous deletion (2%), *CDK4* amplification (18%), *CCND2* (cyclin D2) amplification (2%), *CDK6* amplification (1%), and *RB1* mutation or homozygous deletion (11%) [1]. Alteration of only the Rb pathway is insufficient to induce tumor formation. EGFR amplification enhances the PI3K pro-growth pathway and is typically associated with *CDKN2A* deletions [145,146]. *CDKN2A* loss is associated with the classical subtype of GBM, according to the TCGA study [1].

### 7.4. Ras/MAPK Pathway

#### 7.4.1. NF-1

**Figure 8.**
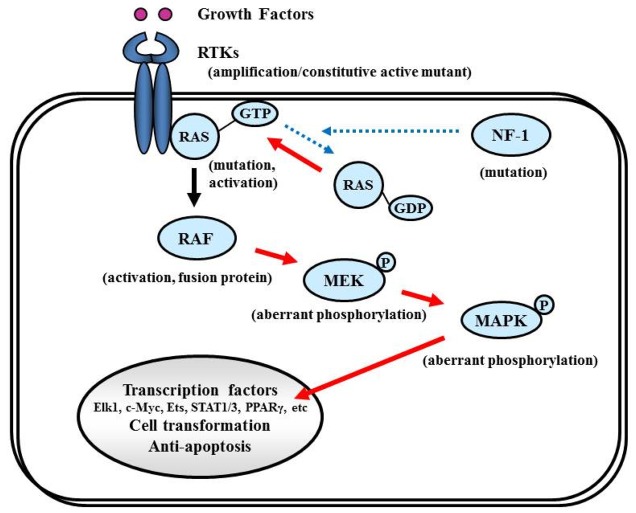
RAS signaling. RAS proteins act as on/off (RAS-GDP/RAS-GTP) switches controlled by RTKs and NF-1. Activated RAS (RAS-GTP) then activates serine/threonine kinase RAF. RAF activates mitogen-activated protein kinase kinase (MAPKK), also called MEK, which in turn activates MAPK. MAPK activation results in activation of various transcription factors, such as Elk1, c-myc, Ets, STAT1/3, and PPARγ, which induce cell transformation and inhibit apoptosis. Solid and dashed arrows indicate activation and suppression, respectively. PPARγ: peroxisome proliferator-activated receptor γ, STAT: signal transducers and activators of transcription.

The NF-1 tumor suppressor gene encodes neurofibromin, which functions primarily as a RAS negative regulator and plays a role in adenylate cyclase- and Akt-mTOR-mediated pathways [147,148] (Figure 8). There is increasing evidence that the NF-1 gene is involved in the tumorigenesis of not only NF-1-related but also sporadically occurring gliomas. In the TCGA pilot study, NF-1 mutation/homozygous deletions were identified in 18% of GBM [1]. Mesenchymal GBMs, having frequent inactivation of the NF-1 (37%), p53 (32%), and PTEN genes, respond to aggressive chemo-radiation adjuvant therapies [90].

#### 7.4.2. RAS

RAS activates the STKs Raf, MAPK (ERK1 and ERK2), PI3K, and a number of proteins that translocate to the nucleus to promote cell proliferation, differentiation, and survival (Figure 8). The *RAS* gene family comprises four different genes that encode HRAS, NRAS, KRAS4A, and KRAS4B [149,150]. Combined activation of RAS and Akt in neural progenitors induces GBM formation in a mouse model [46]. Although activating *RAS* mutations are observed in approximately 30% of human cancers [149], the *RAS* mutations are rare in human GBM (2%, according to the TCGA study [1]). Therefore, the observed deregulation of the Ras/RAF/MAPK signaling pathway in GBM is attributed to its upstream positive regulators, including EGFR and PDGFR, which are highly active in the majority of malignant gliomas [87,151].

#### 7.4.3. RAF

Activated RAS recruits RAF kinase to the membrane, where it is activated by multiple phosphorylation events (Figure 8). The RAF family of STKs, which consist of ARAF, BRAF, and CRAF1, play an important role in proliferative signaling. In pediatric low-grade glioma, consisting of pilocytic astrocytoma and fibrillary astrocytoma, chromosome 7q34 rearrangements result in an in-frame *KIAA1549:BRAF* fusion gene that possesses constitutive BRAF kinase activity resembling oncogenic BRAF (V600E) [152]. In contrast, RAF mutations or rearrangements are rare in malignant gliomas in adults. A phase I/II clinical trial is currently underway for recurrent GBM with combined Raf and mTOR inhibiters, Sorafenib (NexavarR, Bayer, and Onyx, Emeryville, CA, USA) and temsirolimus (CCI-779) (NCT00335764).

#### 7.4.4. MEK/MAPK

Activated RAF phosphorylates and activates MAPK kinase (MAPKK), also called MEK, which in turn phosphorylates and activates MAPK [153]. Activated MAPK translocates into the nucleus to activate several transcription factors such as Elk1, c-myc, Ets, STAT (signal transducers and activators of transcription) 1/3, and PPARγ (peroxisome proliferator-activated receptor γ), which induce cell cycle progression and anti-apoptosis genes [154,155] (Figure 8). Aberrant signaling through this pathway leads to cell transformation and resistance to apoptosis; thus, this pathway is an attractive target for malignant glioma therapy [156].

### 7.5. Glioma Stem Cell (GSC) Pathway

**Figure 9.**
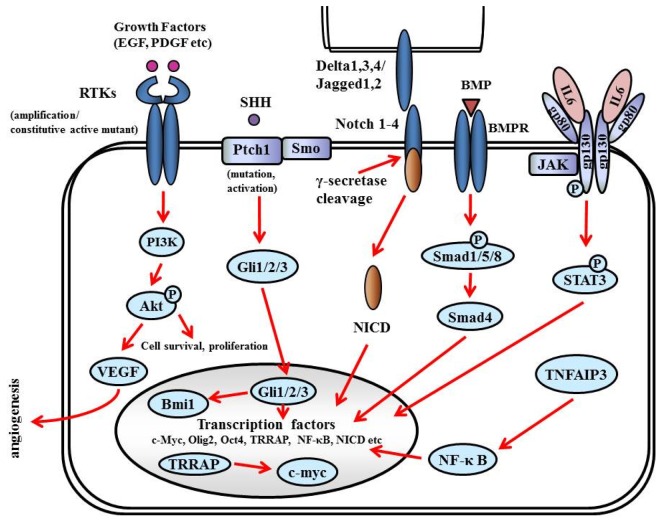
Signaling pathway in glioma stem cell. BMP: bone morphogenetic proteins; BMPR: BMP receptor; Ptch: patched gene; SHH: Sonic hedgehog homolog; STAT: signal transducers and activators of transcription; Smo: smoothened gene; TNFAIP: tumor necrosis factor alpha-induced protein; TRRAP: transformation/transcription domain-associated protein.

GSCs are distinguished by (i) the ability to self-renew, (ii) the ability to initiate brain tumors upon orthotopic implantation in immunodeficient mice, (iii) the expression of neural stem cell markers, and (iv) multipotency—The capacity to differentiate into cells with a neuronal, astrocytic, or oligodendroglial phenotype. GSCs express antigens specific for neural stem and progenitor cells: Nestin, CD133 (prominin-1), Musashi-1, and Bmi-1 [157-159]. CD133^+^ cells comprise 5%–30% of the tumor cell population, and as few as 100 cells can reproduce tumors in animal models [160]. Sonic hedgehog homolog (SHH) and Notch are key regulators of neural progenitors and have been found to be altered or overexpressed in GSCs [161] (Figure 9).

#### 7.5.1. RTKs

RTKs mediate the effects of multiple oncogenic growth factor pathways. Ligand binding to RTK activates PI3K. Activated PI3K activates PDK1 which phosphorylates Akt, which transduces signals for cell survival, proliferation, motility, and angiogenesis as shown in Figure 6. PDGF signaling in neural stem cells is required for oligodendrogenesis, and amplification of this signal induces the aberrant proliferation of neural stem cells and the formation of large glioma-like lesions [162].

#### 7.5.2. SHH

SHH is a critical mitogen for medulloblastoma precursor cells. Hereditary loss of function mutations in the SHH receptor, Patched (PTCH) lead to constitutive activation of the SHH pathway and predisposition to medulloblastoma in Gorlin syndrome. The SHH pathway is also activated in GSCs; the Hedgehog inhibitor cyclopamin depleted clonogenic GBM stem cells *in vitro* and inhibited tumor initiation *in vivo* [163]. The binding of SHH ligands to their receptors activates transducers termed Gli (named for their discovery in gliomas), which translocate into the nucleus and activate (Gli1/2) or repress (Gli3) downstream targets. The SHH pathway is also connected to cell cycling since it inactivates RB1, facilitating the over-expression of cell cycle regulators such as N-myc. Smoothened (SMO) is a downstream protein in the SHH pathway. In the absence of ligand, SMO is inhibited by PTCH1. A current theory suggests that PTCH regulates SMO by removing oxysterols. The binding of SHH relieves SMO inhibition, leading to activation of the GLI transcription factors. Bmi1, a promoter of neural stem cell self-renewal and neural development, is expressed in most gliomas and promotes self-renewal of GSC [164].

#### 7.5.3. Notch

Notch (Notch1-4 in mammals) is a family of transmembrane receptors that regulate intercellular signaling [165]. Notch ligands (Delta-like1, Delta-like3, and Delta-like4, and Jagged1–2 in mammals) are also transmembrane proteins and, when bound to Notch, expose the receptor to proteolytic activation. Presenilins cleave Notch to generate a Notch1 intracellular domain (NICD), which translocates to the nucleus to act as a transcriptional activator. Notch activation induces expression of downstream target genes, such as p53, and promotes neural stem cell growth [166]. The Notch pathway has been targeted using γ-secretase inhibitors (GSIs), depleting CD133-positive GSCs, and inhibiting tumor initiation and growth *in vivo* [167].

#### 7.5.4. Bone Morphogenetic Proteins (BMPs)

BMPs are a family of growth factors named for their central role in bone and cartilage formation. Most BMPs elicit their actions through binding to cell-surface receptor kinases (BMPRs). The effectors of BMPRs are the Smad proteins. Activating phosphorylation of Smad1/5/8 enables these proteins to bind to the co-activator Smad4, translocate into the nucleus, and regulate transcription. BMP ligands deplete the GSC population by inducing the differentiation of GSCs into astroglial and neuron-like cells. Treating GSCs with BMPs *in vivo* delays tumor growth and reduces tumor invasion [168].

#### 7.5.5. IL6

The IL6 signal transduction pathway is initiated by IL6 ligand binding to heteromeric plasma membrane receptor complexes formed from a specific IL6 binding receptor, IL6 receptor α (IL6Rα, gp80), and the common signal transducing receptor glycoprotein 130 (gp130). Upon receptor activation, intracellular signaling is propagated by Janus kinase (JAK) tyrosine kinase family members, leading to activation of transcription factors of the signal transducers and activators of transcription (STAT) family, particularly STAT3 [169]. STAT3 activation, as indicated by phosphorylation at tyrosine705, is present in glioma patient samples and increases with tumor grade [170]. IL6 signals promote STAT3 activation in GBM cells *in vitro*, and targeting either STAT3 or IL6 decreases GBM cell survival [171]. STAT3 is also linked to stem cell biology, as STAT3 is required to maintain pluripotency of normal embryonic stem cells and neural stem cells [172].

#### 7.5.6. Tumor Necrosis Factor alpha-Induced Protein (TNFAIP) 3

TNFAIP3 is a regulator of the NF-κB pathway that is overexpressed in GBM stem cells in comparison to non-stem tumor cells. Knockdown of TNFAIP3 by lentiviral shRNA reduces glioma stem cell self-renewal, growth, and apoptotic resistance [173].

#### 7.5.7. Transcription factors

Transcription factor Olig2 is expressed in oligodendroglia and in mitogen-treated “transit-amplifying cells” of the subventricular zone, the presumed site of most adult neural stem/progenitor cells, and a hypothesized site for the origin of gliomas [162]. Olig2 is almost universally found in NG2-positive glia and is required for development of these cells [174]. NG2 is a chondroitin sulfate proteoglycan that is thought to be another marker of oligodendrocyte progenitor cells. Olig2 promotes the proliferation of both neural progenitors and GBM stem cells by repressing the p21 tumor suppressor [175].

Transformation/Transcription Domain-Associated Protein (TRRAP) is an ATM/R-related pseudokinase that scaffolds several histone acetyltransferase (HAT) complexes. TRRAP plays a central role in c-myc transcription activation; it is required for p53-mediated transcription activation. Knockdown of TRRAP sensitizes GSCs to apoptotic stimuli, decreases self-renewal and proliferation, and suppresses tumor formation *in vivo*, suggesting that TRRAP maintains GSCs in a stem cell-like state [176].

#### 7.5.8. MicroRNA (miRNA)

miRNA is a small non-coding RNA that post-transcriptionally downregulates gene expression during apoptosis, differentiation, and development. Several studies identified aberrant miRNA (microRNA-21, miR-326, microRNA-34a) expression in gliomas, and linked some of them to GSC maintenance and growth [177-179].

## 8. Molecular Targets

Based on the recent progress of molecular biology research in GBM, efforts are currently underway to examine therapeutic targeting of molecules in signaling pathways with the hope of developing effective novel treatments for malignant glioma. Because of their frequent activation or mutation in human GBM and their paramount role in the maintenance of proliferation signaling, both EGFR and PDGFR are plausible targets for molecular therapies. However, clinical trials with drugs targeting EGFR or PDGFR, so far, have yielded disappointing results. However, according to the individual genetic analysis in this clinical trial, the carriers of variant EGFR (EGFRvIII) and wild-type PTEN showed good response to the inhibitors [106]. This example demonstrates why molecular targeting therapies represent a therapeutic strategy of great clinical interest for the treatment of GBM, especially when the inhibitors are rationally selected. So far, VEGF inhibitor (bevacizumab) and integrin inhibitor (cilengitide) have yielded good results in Phase II clinical trials and have moved ahead to Phase III trials.

### 8.1. Bevacizumab

Because of its prominent role in glioma angiogenesis, the VEGF pathway is widely identified as an attractive therapeutic target. Treatment strategies targeting angiogenesis signaling cascades have demonstrated promising results in preclinical studies. In these strategies, targeting the VEGF pathway has yielded the most clinically advanced anti-angiogenic drugs [180]. Several VEGF-targeting approaches are under clinical investigation in malignant gliomas.

One of the most well established anti-angiogenic medicines is bevacizumab, which is an IgG1 monoclonal antibody against free VEGF-A in the circulation, preventing attachment to the VEGF receptor and activation of a pro-angiogenic stimulus. Bevacizumab was originally developed for use in colorectal cancer, and has been approved by the European Medicines Agency for treatment in metastatic breast and kidney cancers [181,182]. Bevacizumab, alone or in combination with chemotherapy, has been associated with reduction in vasogenic brain edema and prolonged progression-free survival. Vredenburgh *et al.* reported the Phase II trial data of recurrent GBM patients who received bevacizumab plus irinotecan every two weeks. The 6-month progression-free survival among all 35 patients was 46%; the 6-month overall survival was 77% [183]. According to the good radiological response and decreasing corticosteroid use, the Food and Drug Administration (FDA) approved bevacizumab in 2009 as a therapy for recurrent GBM [184]. A recent clinical trial reported a 16% 2-year survival rate in recurrent GBM with bevacizumab monotherapy [3]. An international clinical trial is ongoing comparing radiochemotherapy with temozolomide and radiochemotherapy with temozolomide and bevacizumab. Based on the result, bevacizumab may be added as the standard therapy. However, recent data suggest that not all vessel formation in glioma depends on VEGF; non-endothelial cell-lined blood vessels are formed by tumor cells, indicating that some GBMs may grow in the absence of endothelial cell recruitment [185,186].

Although the response rate to anti-angiogenic agents targeting VEGF for GBM is 40%–60%, effective responses are commonly transient. Some glioma patients may exhibit primary refractoriness to the agents targeting VEGF. As yet, anti-angiogenic therapy is one of the most effective treatments for glioma patients; further understanding of the mechanisms of resistance to anti-angiogenic therapies and better patient selection will be crucial to improve outcomes for patients with GBM.

### 8.2. Cilengitide

The integrin family of cell adhesion receptors is emerging as a promising target of anticancer therapy. α3β1, αvβ1, αvβ3, and αvβ5 integrins are overexpressed on both glioma cells and tumor vasculature. Cilengitide, the most advanced specific αvβ3 and αvβ5 integrin inhibitor in oncology, has shown antitumor activity against glioma in early clinical trials.

Obvious benefits have been observed in phase I and phase II trials with cilengitide. A phase II clinical trial using cilengitide for recurrent GBM reported 23% 2-year survival and 10% 4-year survival [3]. In the clinical trial using cilengitide in conjunction with standard chemoradiotherapy for newly diagnosed GBM, progression-free survival was eight months and overall survival was 16.1 months [187]. Cilengitide is now being explored in prospective randomized phase III (CENTRIC) trials in the first-line setting in combination with standard radiochemotherapy.

### 8.3. Multi-Kinase Inhibitors

Multiple RTKs are concomitantly activated in GBMs *in vitro* and *in vivo*. Redundant inputs drive and maintain downstream signaling [188]. This finding can explain the weak clinical responses to RTK-inhibitor monotherapy. There are commonly multiple target molecules in individual GBM. Multi-kinase inhibitors are being developed with the expectation of more favorable outcomes against GBM. Dasatinib is an inhibitor targeting c-Src, c-kit, PDGFR, and Bcr-Abl. Enzastaurin is an STK inhibitor that targets protein kinase C and PI3K/Akt. Both agents are now in clinical trials.

## 9. Conclusions and Perspectives

Recent studies have highlighted the critical pathological roles played by various signaling networks and complex signaling crosstalk in the progression of glioma. It is important to clarify the dominant signaling pathways and crosstalk in order to establish effective molecular targeted therapies against GBM.

Future studies are necessary to delineate the signaling elements implicated in the positive and negative regulation of the malignant phenotype of GBM. In particular, it will be important to identify the factors involved in the enhancement of oncogenic signaling and potential interactive crosstalk with other oncogenic pathways during glioma progression. These studies should lead to the identification of new potential targets, optimized choice of therapeutic regimens, and personalized medicine, as well as the development of novel effective molecular targeted strategies that could be used to improve current GBM therapies.

The number of clinical trials for GBM patients will increase as new targets are discovered. Most importantly, well-designed clinical trials should be planned because the incidence of GBM is relatively low compared with other carcinomas. Furthermore, surgical tissue should be accessed at relevant time points to analyze pathway activation biomarkers. Ideally, GBM will be categorized by its molecular profile, to identify patients who may be expected to respond to certain molecular targeted drugs will be appropriately selected for clinical trials. In the future, the concept of “tailor-made molecular targeted therapy” against GBM will become reality.

## Figures and Tables

**Table 1.** Major angiogenic factors in malignant gliomas.

**Angiogenic factors**	**Function**
VEGF-A, -B, -C and -D	Angiogenesis, promotes endothelial proliferation and migration, mitosis of endothelial cells and creation of blood vessel lumen
VEGFR1	Hematopoiesis, promotes tumor angiogenesis, participates in the activation of MMP
VEGFR2	Mediates the mitogenic, angiogenic and permeability-enhancing effects of VEGF-A
Angiopoietin	Induces stabilization, remodeling and maturation of blood vessels
Acidic FGF and basic FGF	Resists apoptosis of endothelial cells, induces endothelial cell proliferation and migration
PDGF	Recruits of pericytes and facilitates migration of pericytes into newly formed blood vessels
EGF	Stimulates VEGF production in glioma cells
TGF-β	Participates in regulator of proliferation, migration, differentiation and ECM synthesis in endothelial cells
SF/HGF	Promotes tumor growth and angiogenesis *in vivo*
IL-6	Induces transcriptional activation of VEGF and regulates VEGF promoter activity
IL-8	Exerts potent angiogenic properties on endothelial cells via the interaction with the CXC chemokine receptor 1 and 2
TNF-α	Involved in systemic inflammation and acute phase reaction, induces tubular morphogenesis *in vitro*
IGF-1	Involved in immunoreactivity in the tumor cells, promotes microvascular proliferation
Integrins	Responsible for the interaction of endothelial and tumor cells with ECM
MMP-2 and MMP-9	Involved in the proteolytic degradation of ECM components and facilitate cell motility during invasion and angiogenesis

HGF: hepatocyte growth factor; IGF, insulin-like growth factor; IL: interleukin; MMP: matrix metalloproteinase; PDGF: platelet-derived growth factor; SF: scatter factor; TGF: transforming growth factor; TNF: tumor necrosis factor; VEGF: vascular endothelial growth factor.
